# Investigation of Key Parameters of Rock Cracking Using the Expansion of Vermiculite Materials

**DOI:** 10.3390/ma8105351

**Published:** 2015-10-12

**Authors:** Chi-Hyung Ahn, Jong Wan Hu

**Affiliations:** 1 Korea Railroad Research Institute (KRRI), Uiwang-si, Gyeonggi-do 437-757, Korea; chahn@krri.re.kr; 2 Department of Civil and Environmental Engineering, Incheon National University, Incheon 406-840, Korea; 3 Incheon Disaster Prevention Research Center, Incheon National University, Incheon 406-840, Korea

**Keywords:** key parameters, rock cracking, thermal expansion, vermiculite

## Abstract

The demand for the development of underground spaces has been sharply increased in lieu of saturated ground spaces because the residents of cities have steadily increased since the 1980s. The traditional widely used excavation methods (*i.e.*, explosion and shield) have caused many problems, such as noise, vibration, extended schedule, and increased costs. The vibration-free (and explosion-free) excavation method has currently attracted attention in the construction site because of the advantage of definitively solving these issues. For such reason, a new excavation method that utilizes the expansion of vermiculite with relatively fewer defects is proposed in this study. In general, vermiculite materials are rapidly expanded in volume when they receive thermal energy. Expansion pressure can be produced by thermal expansion of vermiculite in a steel tube, and measured by laboratory tests. The experimental tests are performed with various influencing parameters in an effort to seek the optimal condition to effectively increase expansion pressure at the same temperature. Then, calibrated expansion pressure is estimated, and compared to each model. After analyzing test results for expansion pressure, it is verified that vermiculite expanded by heat can provide enough internal pressure to break hard rock during tunneling work.

## 1. Introduction

The urban area has been rapidly increased in the world, and thus underground space used for territorial expansion has been excavated instead of ground space that has reached a saturation point. The development of underground space has actively proceeded in order to secure the commercial district, transportation space, and residential area. Many engineers recognize that the development of underground spaces is relatively more economic and effective than the exploration of the ocean and outer space. The excavation of the tunnel has been considered one of the most feasible ways to extend underground space, and is classified into either the mechanical method utilizing massive scale equipment (e.g., shield and hydraulic machinery) or the blasting method using dynamite [1,2]. Although the blasting method is faster and more economical when compared to the mechanical method, it may result in serious troubles caused by noise and vibration, which may severely damage structures on the ground [3,4]. On the other hand, the mechanical method used for excavating a tunnel is quite capable of reducing noise and vibration during construction [5]. However, it is required to not only to manage huge equipment but it also has high maintenance costs. This method is more prone to waste because of the extended construction period [6,7]. To overcome drawbacks resulting from these traditional excavation methods, many scientists have launched studies with regard to the vibration-free excavation method, and devoting themselves to the application at construction sites [2,8,9]. Despite the many advantages with respect to simple equipment, quick construction, and being economical, the vibration-free excavation method using rock breakage by thermal expansion still remains at the early stage of development when compared to the existing methods. Furthermore, failure mechanisms, dynamic mechanical properties, and deformation fields for rock mass should be fully obtained based on exact measurement techniques in order to raise the effectiveness of the vibration-free evacuation method [10]. However, traditional contact measurement techniques, such as electrical resistance strain gauges and mechanical extensometer, possess a lot of limitations in measuring range and frequency responses, and accordingly cannot provide adequate information to address the complexity of dynamic mechanical behavior. For these reasons, previous research related to developing new vibration-free excavation methods has faced many difficulties in effectively cracking hard rocks.

For these motivations, this study suggests a new vibration-free (and also explosion-free) excavation method that utilizes thermal expansion materials used for splitting rock, and also intends to verify technological efficiency and excellence through laboratory experiments. Simple cartridge heaters, which are able to shift electrical energy into thermal energy, are installed in cylindrical-shaped rock excavation holes, and then gaps between cartridge heaters and excavation holes are filled with thermal expansion materials. The rock can be spilt by expansion pressure into several parts when excessive heat radiating from heater devices is applied to the thermal expansion materials. Cost-efficient and light vermiculite materials are used for thermal expansion in this study. Vermiculite, when heated, can expand up to 20 times, thereby generating considerable high pressure. The compound medium for thermal conduction (e.g., silicon carbide, SiC) has to be added with the same mix ratio because vermiculite itself acts as only heat insulation. The excavation method proposed herein makes good use of simple equipment, low cost materials, as well as easy field application, and thus minimizes not only construction costs but also labor costs. In addition, it can mitigate the issues of noise and vibration because there are no explosions. In an effort to adequately apply this new vibration-free excavation method to a construction field, investigation was conducted to seek the optimized condition that will maximize expansion pressure by regulating key influence parameters through a series of laboratory experiments. After changing the vermiculite particle size, mixing ratio, and heating condition, expansion pressure will be measured, while increasing the temperature steadily during the experimental test. The maximum expansion pressures are compared to each model in a bid to present the optimal use of vermiculite.

## 2. Vermiculite Expansion

Vermiculite can be expanded by up to 20 times in volume when heated rapidly [11]. This happens in the form of exfoliation process caused by an explosive release of interlayer water. There are large commercial vermiculite mines in Russia, South Africa, China, and Brazil. The vermiculite in an exfoliated (or expanded) state can raise industrial interest because it is a non-toxic, lightweight, fire-resistant, absorbent, and odorless material. These properties permit vermiculite to be utilized in various applications such as environmental protection, attic insulation, building materials, and the chemical industry [11]. The size of vermiculite products ranges from very fine particles to large (coarse) pieces that reach nearly an inch long. In general, industrial vermiculite is a kind of aggregate made up of vermiculite mixed with a layer of minerals, and not pure vermiculite [11,12].

**Figure 1:**
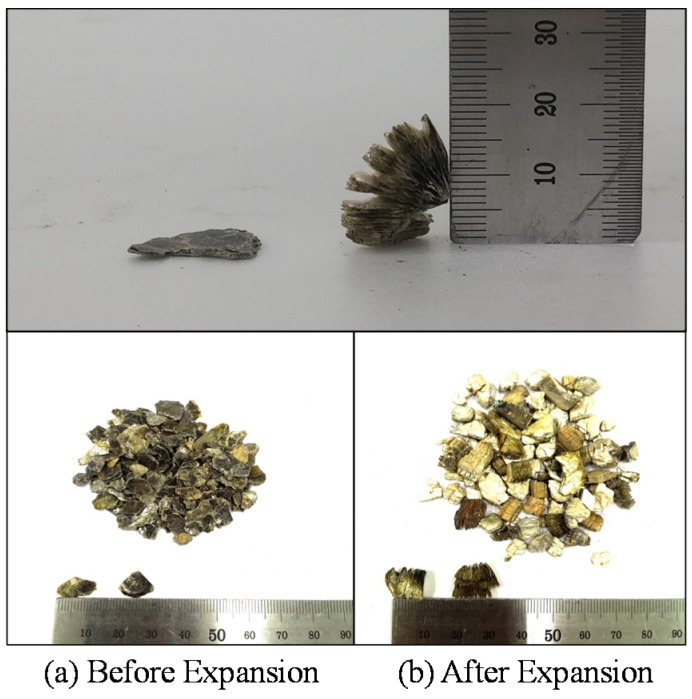
Thermal expansion of the vermiculite materials.

The expansion of the vermiculite material compared to the original configuration before expansion is presented in Figure 1. The expansion of vermiculite is mainly obtained through three methods: (1) chemical expansion method, (2) microwave expansion method, and (3) thermal expansion method [13,14]. For the chemical expansion method, hydrogen peroxide solution is the most commonly used to exfoliate phyllosilicate since it has the best expansion effect among activated regents. Once hydrogen peroxide solution is impregnated into vermiculite, it will exchange with interlayer water molecules. Thereafter, thes interlayer water molecules can decompose the layer of vermiculite so that vermiculite will expand [14]. As far as the microwave expansion method is concerned, microwaves vibrate water molecules infiltrated into the interlayer of vermiculite with high frequency, and then water molecules collide with each other to generate friction heat. In this method, the expansion of vermiculite is mainly affected by this friction heat. Finally, the thermal expansion method directly applies heat to vermiculite so that interlayer water quickly converts into steam. Formed vaporization pressure exfoliates immediately from the interlayer of the vermiculite. This thermal expansion method is the most effective compared to the other two expansion methods because heat is able to vaporize interlayer water molecules directly applied to vermiculite. This study uses the thermal expansion method in order to obtain safe and good expansibility using heater equipment.

## 3. Experimental Tests

### 3.1. Experimental Setup

**Figure 2:**
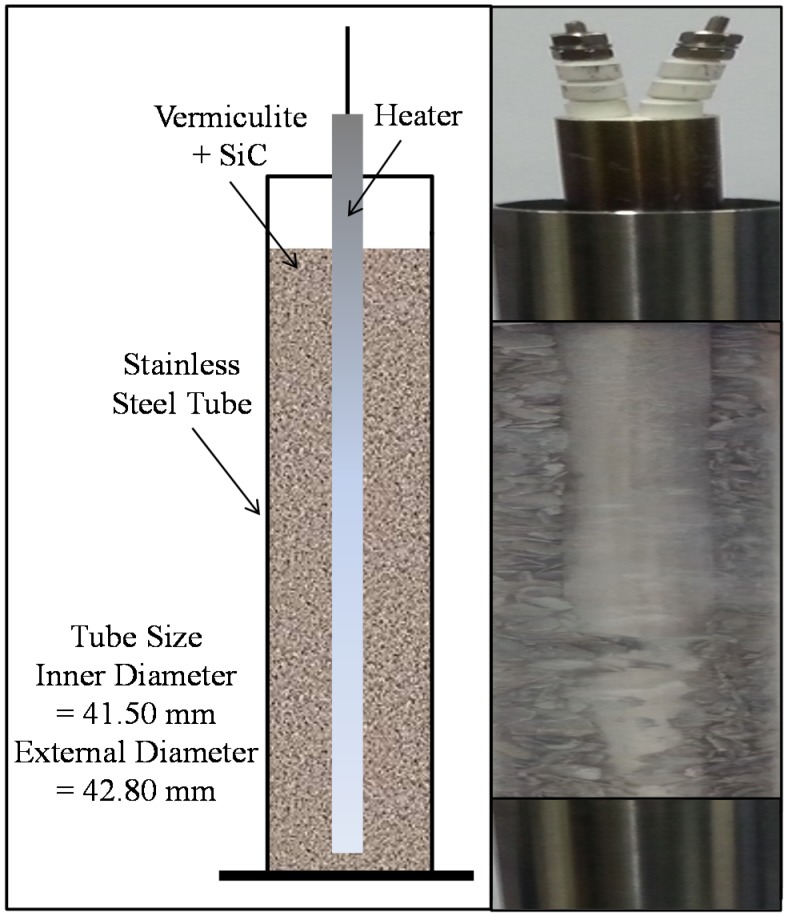
Stainless steel tube filled with vermiculite, silicon carbide (SiC) and cartridge heater.

Instruments for the experimental tests involved with the measurement of the expanded tube, are firstly introduced, and then their manipulations are mainly treated in this section. The extent of expansion and pressure varies in accordance with parameters such as vermiculite particle size, mixture ratio, and heating condition. For laboratory tests, stainless steel tubes are used to measure radial expansion accurately instead of using natural rocks. The core instruments for the laboratory test are presented in Figure 2. The stainless steel tube was filled with vermiculite, silicon carbide (SiC), and cartridge heater. Silicon carbide as a heat conductor should be equally mixed with vermiculite, which has insulation characteristics, in order to pass heat to vermiculite particles thoroughly. The requirement for estimating expansion pressure is that the stainless steel tubes remain elastic without plastic deformation. Young’s modulus, Poisson’s ratio, and yield stress of typical stainless steel tubes are taken as 193 GPa, 0.3 and 345 MPa, respectively. The stainless steel tubes used here were fabricated with 41.5 mm inner diameter and 42.8 mm outer diameter.

The parameters that have an influence on the extent of expansion pressure on the stainless steel tube are applied to nine test models individually, as summarized in Table 1. As far as vermiculite particle size is concerned, T1 series models can be classified into three models, T1-1 model for large particle size (8 mm), T1-2 model for middle particle size (5 mm), and T1-3 model for small particle size (3 mm). For particle mixing ratios, T2 series models can also be classified into three model cases presented in Table 1. T1 and T2 series models are heated by 21 mm diameter cartridge heaters. T3 series models can be classified in accordance of the diameter of heater apparatus such as T3-1 model for 24 mm diameter heater, T3-2 model for 21 mm diameter heater, and T3-3 model for 18 mm diameter heater. T3 series models are designed with the same particle mixing ratio (*i.e.*, Large Particle Size (LPS): Middle Particle Size (MPS) = 9:1). Finally, all test models are fabricated with equal mixing ratios by weight between vermiculite and silicon carbide.

**Table 1:** Individual models for experimental tests.

Model ID	Conditions	Other Remarks
T1-1	Large Particle Size: LPS (8 mm)	Diameter of Heater (21 mm) **Equal Mixing Ratios
T1-2	Middle Particle Size: MPS (5 mm)	
T1-3	Small Particle Size: SPS (3 mm)	
T2-1	*Particle Mixing Ratio (LPS:MPS = 9:1)	Diameter of Heater (21 mm) **Equal Mixing Ratios
T2-2	*Particle Mixing Ratio (LPS:MPS = 7:3)	
T2-3	*Particle Mixing Ratio (LPS:MPS = 5:5)	
T3-1	Diameter of Heater (24 mm)	*Particle Mixing Ratio (LPS:MPS = 9:1) **Equal Mixing Ratios
T3-2	Diameter of Heater (21 mm)	
T3-3	Diameter of Heater (18 mm)	

* Particle mixing ratio by weight between vermiculite materials. **All test models are fabricated with equal mixing ratios by weight between vermiculite and silicon carbide.

**Figure 3:**
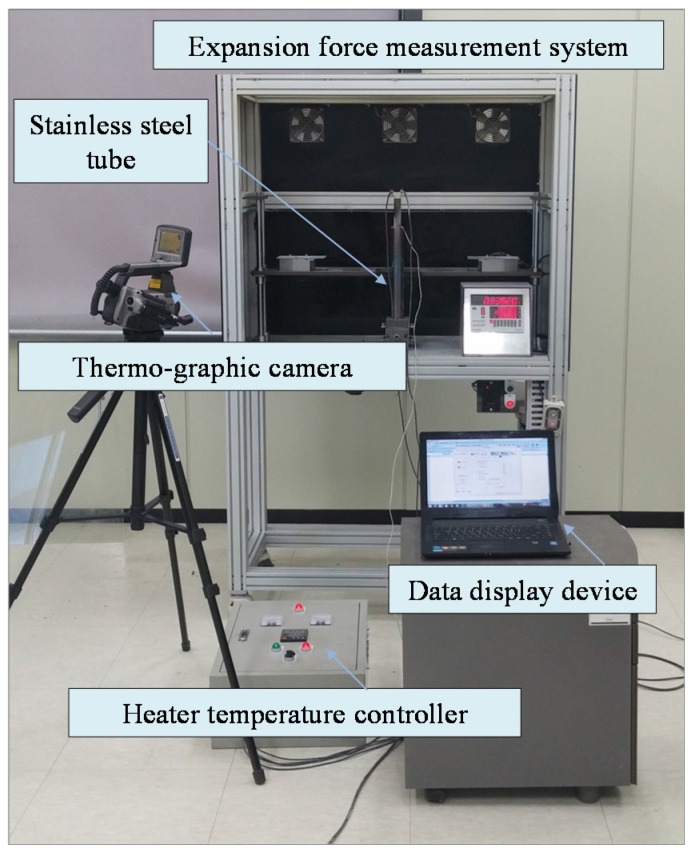
Instrumentation of laboratory equipment.

The instrumentation of laboratory equipment for the experimental test, including the stainless steel tube, is presented in Figure 3. The cartridge heater inserted into the stainless steel tube was connected to a controller capable of regulating the temperature of the heater. Once silicon carbide has been conducted with heat generated by the cartridge heater, vermiculite mixed with the silicon carbide begins to expand inside the stainless steel tube. The expanded vermiculite volume can uniformly radiate internal pressure toward the radial direction, and consequently internal pressure was estimated by measuring the displacement of the stainless steel tube, which was expanded along the radial direction. The expansion of the stainless steel tube can be monitored at time and temperature on the data display device. Except for expansion caused by the change of vermiculite volume, surplus heat conducted to the stainless steel tube has a direct influence on radial expansion. For this reason, it is necessary that the amount of radial expansion should be calibrated by eliminating thermal expansion on the stainless steel tube itself. The FLIR ThermaCAM S65 infrared camera (YELLOWTEC Company, Gauteng, South Africa) [15] was used to detect the thermal imaging contour and to measure the temperature on the surface of the stainless steel tube. This thermos-graphic camera can measure temperature ranging from –40 and 1500 °C with the reading accuracy of ± 2 °C. The calibration can be achieved by automatic corrections based on user input for reflected ambient temperature, distance, relative humidity, atmospheric transmission, and external optics. The spectral range of the camera is between 7.5 and 13 μm. The high-resolution 14-bit thermal images in real time can be achieved by thermal sensitivity of 0.08 °C coupled with a 76,000 pixel display. The focal plane array (FPA) uncooled micro-bolometer was used as a special detector.

**Figure 4:**
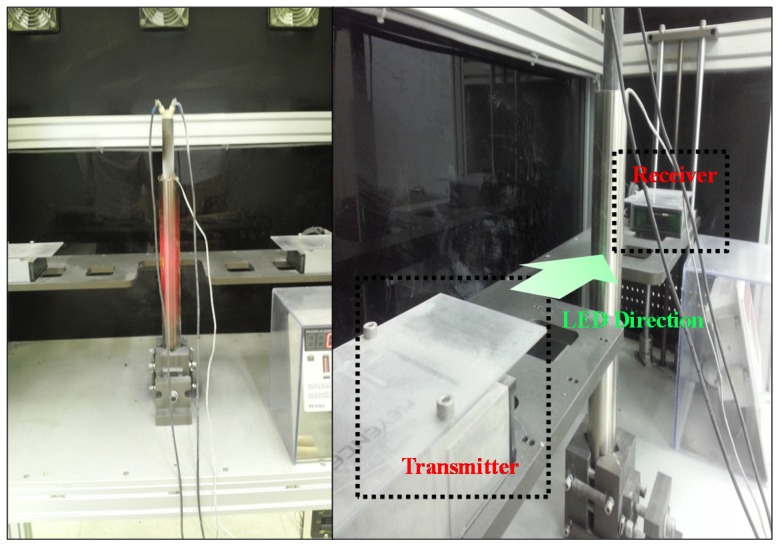
Measurement of thermal expansion.

As seen in Figure 4, the LS-9000 sensor made by the KEYENCE Company (Osaka, Japan) was used to measure the extent of thermal expansion, as shown in Figure 4. This measurement sensor consists of transmitter and receiver devices. The receiver takes the LED beam that the transmitter sends out. As the diameter of the stainless steel tube increases due to thermal expansion, the band of the LED beam decreases. The receiver detects the size of the LED beam band, and thus the amount of radial expansion is calculated by measuring clearance between the surface of the expanded tube and the edge of the LED beam. The reference temperature of the tube surface was set at 20 °C (T_0_ = 20 °C). Both surface temperature and expansion size were measured until the cartridge heater arrived at the temperature of 900 °C.

### 3.2. Expansion Measurements

**Figure 5:**
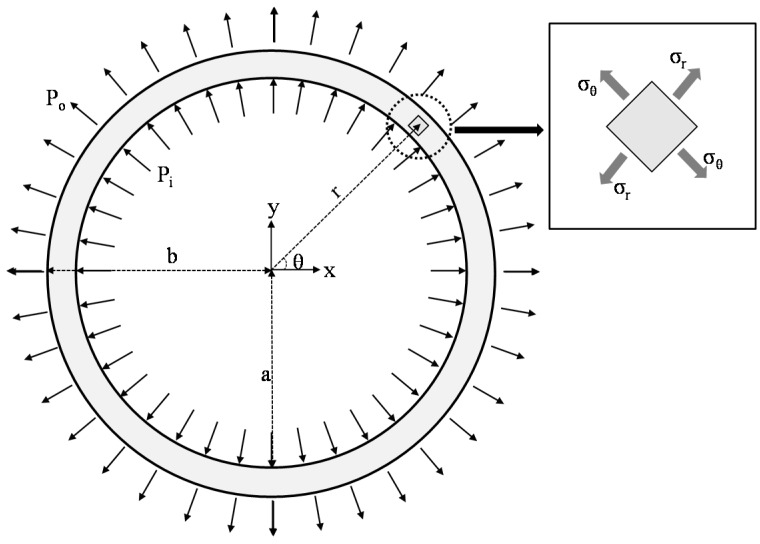
Stress components for the thin walled pressure tube (vessel).

The stainless steel tube can be considered to be a thin-walled cylindrical pressure vessel so as to measure its expansion along the radial direction. As can be seen in Figure 5, there are two stress components for the thin-walled tube subjected to internal or external uniform pressure (*P_i_* or *P_o_*), such that ${\text{σ}}_{\theta }$ is the tangential stress and ${\text{σ}}_{r}$ is the radial stress [16]. Before calculating the stress components, it is necessary to check whether stainless steel tubes are designed to satisfy the limit condition of the thin-wall pressure vessel or not, as follows:

(1) $$\frac{b-a}{a}\geq \frac{1}{20}$$

where, *a* and *b* denote the inner and outer radius of the thin-walled cylindrical pressure vessel, respectively. Radial stress is equal to −*P_i_* on the inner surface, +*P_o_* on the outer surface, and varies in between two surfaces as follows [16]:

(2) $${\text{σ}}_{r}=\frac{\left(a^{2}P_{i}+b^{2}P_{o}\right)}{\left(b^{2}-a^{2}\right)}-\frac{a^{2}b^{2}\left(P_{i}+P_{o}\right)}{r^{2}\left(b^{2}-a^{2}\right)}$$

For another principle stress, tangential stress can be derived by considering Mohr’s circle as follows [16]:

(3) $${\text{σ}}_{\theta }=\frac{\left(a^{2}P_{i}+b^{2}P_{o}\right)}{\left(b^{2}-a^{2}\right)}+\frac{a^{2}b^{2}\left(P_{i}+P_{o}\right)}{r^{2}\left(b^{2}-a^{2}\right)}$$

The radial displacement of the thin-walled cylindrical pressure vessel is also derived by taking Hook’s law into consideration as follows:

(4) $$u_{r}=\frac{1-\upsilon }{E}\frac{\left(a^{2}P_{i}+b^{2}P_{o}\right)}{r\left(b^{2}-a^{2}\right)}+\frac{1+\upsilon }{E}\frac{a^{2}b^{2}\left(P_{i}+P_{o}\right)}{r\left(b^{2}-a^{2}\right)}$$

where, *E* and *υ* indicate Young’s modulus and Poisson’s ratio of the stainless steel tube, respectively [16].

The thin-walled cylindrical pressure vessel treated in this study belongs to the case where only internal pressure exists (e.g., *P_o_* = 0), and thus the equations mentioned above can be rewritten as follows [16]:

(5) $${\text{σ}}_{r}=\frac{a^{2}P_{i}}{\left(b^{2}-a^{2}\right)}\left(1-\frac{b^{2}}{r^{2}}\right)$$

(6) $${\text{σ}}_{\theta }=\frac{a^{2}P_{i}}{\left(b^{2}-a^{2}\right)}\left(1+\frac{b^{2}}{r^{2}}\right)$$

(7) $$u_{r}=\frac{P_{i}a^{2}r}{E\left(b^{2}-a^{2}\right)}\left(\left(1-\upsilon \right)+\left(1+\upsilon \right)\frac{b^{2}}{r^{2}}\right)$$

The maximum radial stress occurs on the inner surface (*r* = *a*) while the maximum radial displacement occurs on the outer surface (*r* = *a*) as follows:

(8) $${\text{σ}}_{r=a}=-P_{i}$$

(9) $$u_{r=b}=\frac{2P_{i}a^{2}b}{E\left(b^{2}-a^{2}\right)}$$

Finally, internal pressure to extend the stainless steel tube along the radial direction can be derived by converting Equation (9) as follows:

(10) $$P_{i}=\frac{E\left(b^{2}-a^{2}\right)}{2a^{2}b}u_{r=b}$$

After measuring the radial expansion of the stainless tube, expansion pressure will be estimated by using Equation (10).

## 4. Experimental Results

As temperatures increased, from heat provided by the cartridge heater, the diameter of the stainless steel tube was measured in real time with an aim to detect expanded radial displacement. The stainless steel tube was expanded along the radial direction owing to expansion not only by the change of the vermiculite volume but also by the change of the tube surface temperature. In this study, only the effect of vermiculite expansion should be taken into consideration for estimating internal pressure. Therefore, it is necessary for the radial displacement measured on the outer tube surface (*r* = *b*) to be calibrated by subtracting average thermal expansion on the stainless steel tube. The radial displacement expanded by temperature change (*T − T_o_*) is defined as follows:

(11) $$u_{t}=b\text{α}\left(T-T_{o}\right)$$

The thermal expansion coefficient of the typical stainless steel (α) is taken as 15.6 × 10^−6^ °C.

Finally, internal pressure calibrated by removing the effect of thermal expansion of the stainless is estimated by the equation below.

(12) $$P_{i}=\frac{E\left(b^{2}-a^{2}\right)}{2a^{2}b}\left(u_{r=b}-u_{t}\right)$$

**Figure 6:**
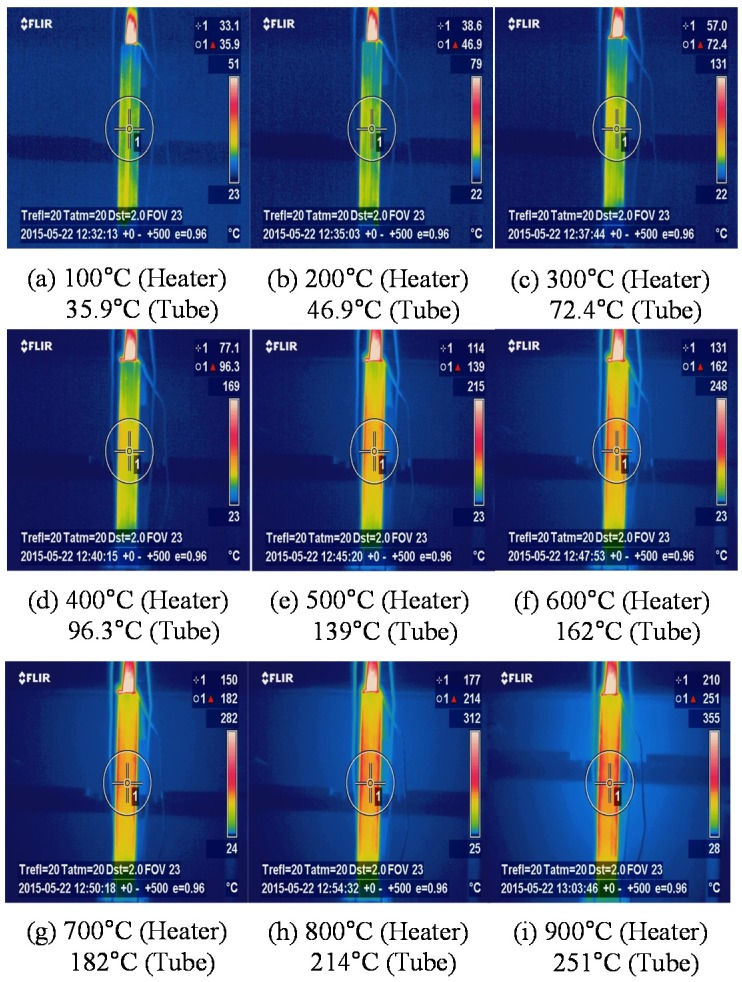
Thermal imaging contours captured by the thermo-graphic camera (T3-1 Model). (**a**) 100 °C heater (**b**) 200 °C heater (**c**) 300 °C heater (**d**) 400 °C heater (**e**) 500 °C heater (**f**) 600 °C heater (**g**) 700 °C heater (**h**) 800 °C heater (**i**) 900 °C heater.

**Figure 7:**
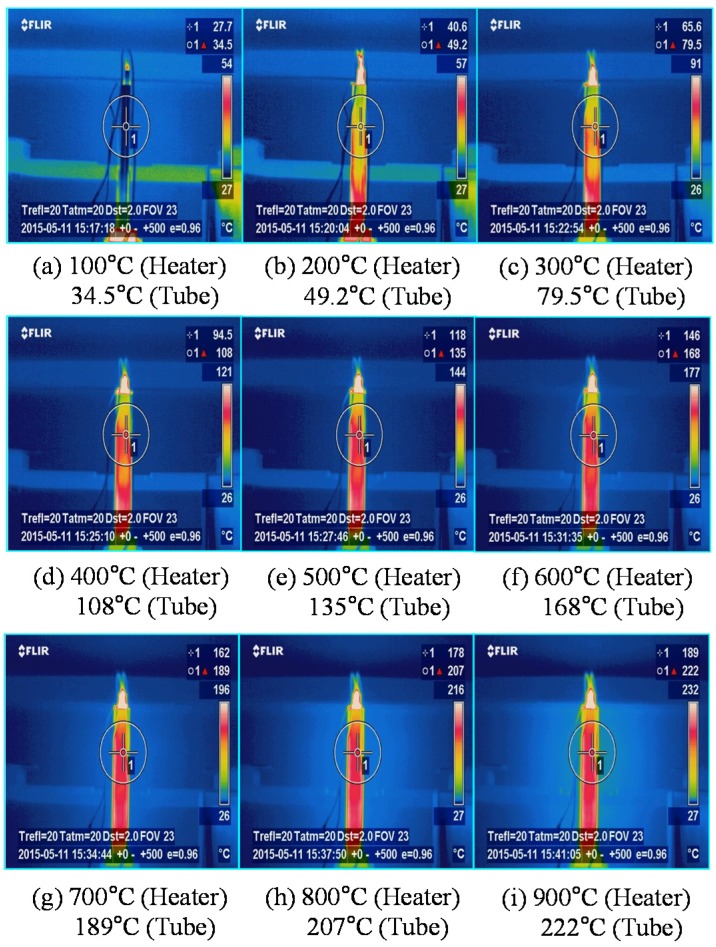
Thermal imaging contours captured by the thermo-graphic camera (T3-2 Model). (**a**) 100 °C heater (**b**) 200 °C heater (**c**) 300 °C heater (**d**) 400 °C heater (**e**) 500 °C heater (**f**) 600 °C heater (**g**) 700 °C heater (**h**) 800 °C heater (**i**) 900 °C heater.

Thermal imaging contours captured by the thermos-graphic camera are illustrated in Figure 6 and Figure 7, which are distributed over T3-1 model and T3-2 model, respectively. As the thermal heat given by the cartridge heater increases, the temperature of the stainless steel tube steadily increases as well. The melting point of the stainless steel tube, referred to as the temperature where a substance changes from solid state to liquid state, is at least 1500 °C, exceeding greatly the maximum captured temperatures. Furthermore, thermal stress at the maximum temperature is far below yield stress for both stainless steel tubes (345 MPa), and thus remains elastic all throughout the experimental test. Accordingly, it is possible to apply the superposition theorem to the calibration process without problems.

**Figure 8:**
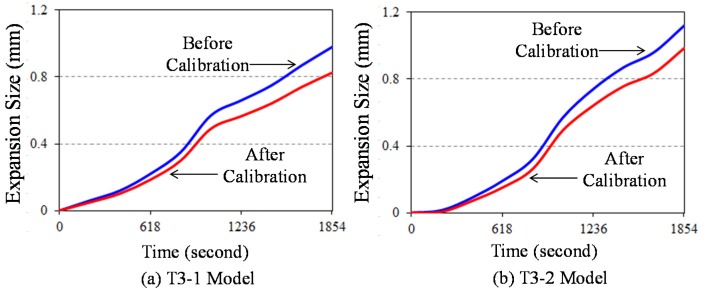
Time *versus* expansion size resulting curves.

As time passes, the resulting curves for the expansion of the stainless steel tubes along the radial direction are presented in Figure 8. There was an approximately 29 °C increased every 60 s. In the figure, two resulting curves before and after calibration were compared. As mentioned above, the resulting curve after calibration excludes the effect of thermal expansion of the stainless steel tube itself (see Figure 6 and Figure 7) from originally measured expansion size. The discrepancy of expansion size between before and after calibration gradually increases over time. The velocity of vermiculite expansion, which is referred to as the slope of the resulting curve, accelerates after about 700 s (equivalently 350 °C). Generally, the T3-2 model with a smaller heater size shows larger expansion size under the same time than the T3-1 model. This is because the T3-2 model contains more vermiculite volume occupied by the larger clearance space between heater and inner tube surface when compared to T3-1 model. The resulting curve of T3-2 model after calibration reaches approximately 1.0 mm expansion size, while that of T3-2 model after calibration arrives at 0.8 mm expansion size. The resulting curves for radial expansion can be converted to those for internal expansion pressure by means of Equation (12).

## 5. Parametric Investigation

**Figure 9:**
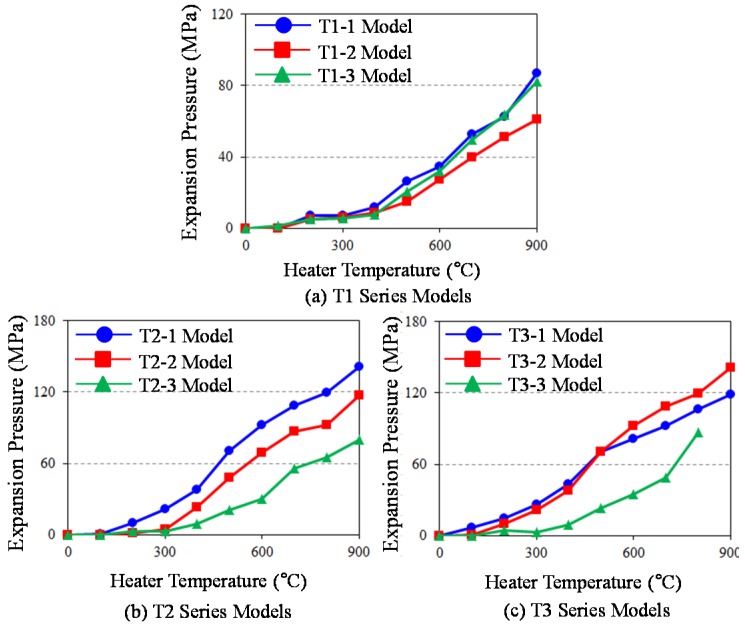
Temperature *versus* calibrated expansion pressure curves.

Relationships between expansion pressure and the abovementioned key parameters are investigated to steadily increase the temperature of the cartridge heater. According to series models distinguished by individual distinct parameters (refer to Table 1), temperature *versus* calibrated expansion pressure curves are presented in Figure 9. T1 series models presented in Figure 9 predicts a slow increase in expansion pressure until temperature arrives at about 350 °C. Beyond this temperature point, T1-1 model with large vermiculite particle sizes exhibits the fastest growth for expansion pressure, and T1-3 model immediately follows. Both T1-1 and T1-3 models exceed 80 MPa expansion pressure at the temperature of 900 °C, meaning that large or small vermiculite particle sizes are more effective for elevating expansion pressure quickly. The common hard rocks found in a tunnel are granite and marble. A piece of granite and marble begins cracking when tensile and flexural stress exceeds their strength limits, 9 MPa for tensile strength and 13 MPa for flexural strength. It can be confirmed that all of the T1 series models should break granite and marble at a temperature of, at most, 500 °C.

As far as T2 series models are concerned, T2-1 model shows the fastest increment of expansion pressure among them at the same temperature, which is attributable to middle-sized vermiculite particles that effectively fill the voids created by large-sized vermiculite particles at the presented particle-mixing ratio (Large Particle Size (LPS):Middle Particle Size (MPS) = 9:1, see Table 1). Overall, T2 series models have larger expansion pressure than T1 series models under the same temperature condition. In particular, T2-1 model is able to break a piece of granite, even at a temperature of 300 °C. For T3 series models, T3-2 model with 21 mm diameter heater is the most appropriate case, increasing the increment of expansion pressure very quickly. The T3-3 model, with relatively smaller 18 mm diameter heater, experimental tests were stopped at 800 °C, and expansion pressure rapidly deteriorated when compared to other two models. Finally, it can be concluded that, based on analyzing these results, the model with the following experimental conditions achieve better efficiency for increasing expansion pressure under the same temperature: particle mixing ratio, LPS:MPS = 9:1; and 21 mm heater diameter. Aside from this, all models are able to generate expansion pressure adequate for cracking a diversity of hard rocks at temperatures far below 900 °C.

## 6. Concluding Remarks

The new vibration-free and explosion-free excavation method used for rock breakage only by expansion pressure is proposed in this study. Once typical vermiculite materials undergo temperature change, they are likely to expand their volume. Owing to these unique characteristics, internal expansion pressure in the stainless steel tube is easy to generate by only applying heat to vermiculite equally mixed with silicon carbide. Calibrated radial displacement was measured through a laboratory experiment performed on stainless steel tubes with various parametric conditions, and then expansion pressure was calculated based on mechanical equations for the thin-walled cylindrical pressure vessel. The key parameters for influencing the increment of expansion pressure were mainly investigated after completing experimental tests. For T1 series models, the models with relatively large or small vermiculite particle size accelerated expansion pressure at the same temperature. For T2 series models, minimizing voids between vermiculite particles is necessary to rapidly raise expansion pressure by mixing each size of vermiculite particle at an adequate mixing ratio. For T3 series models, it is necessary to select a cartridge heater diameter with the aim of securing adequate clearance. In this study, T3-2 model with 21 mm heater diameter showed the best efficiency in elevating expansion pressure at the same temperature. After estimating expansion pressure for each of the model cases, it is confirmed that vermiculite expansion occurring in a confined space provides enough force to split hard rock. All of the model cases presented herein exceeded the tensile and flexural strength limit for marble and granite rocks.
